# Do penile cutting practices other than full circumcision protect against HIV?

**DOI:** 10.1186/1471-2334-14-S2-P17

**Published:** 2014-05-23

**Authors:** John M Kaldor, David MacLaren, John McBride, Peter Siba, Andrew Vallely

**Affiliations:** 1 Kirby Institute, University of New South Wales, New South Wales, Sydney, Australia; 2 James Cook University, Townsville City, Queensland, Australia; 3 Papua New Guinea Research Center, Goroka, Papua New Guinea

## Introduction

Male circumcision provides a high level of protection against sexually acquired HIV infection and is a key element of prevention in countries with extensive heterosexual transmission. In some countries, penile cutting practices other than full circumcision are a part of the cultural landscape, raising the question of their ability to modify the risk of HIV. One such country is Papua New Guinea.

## Methods

We reviewed information on prevalence of HIV, sexually transmitted infection (STI), and penile cutting practices, and their possible relationships.

## Results

Based on antenatal testing, the prevalence of HIV infection among pregnant women in Papua New Guinea is around 0.9%. Surveys of STI in pregnant women have found prevalences of chlamydia, gonorrhoea and trichomonas in the range 15-25%, and infectious syphilis at 2-3%. In three studies of penile cutting around half the men have some form of procedure; 10% had full foreskin removal with a further 30-40% having dorsal slits, with lateral retraction of the foreskin and exposure of the glans. There is evidence of an inverse geographic correlation between HIV prevalence and partial cutting practice.

## Conclusions

Levels of curable STIs in Papua New Guinea are very high by international levels, while HIV infection is at moderate levels compared to the countries in which male circumcision is now being promoted. The role of partial penile cutting procedures deserves further examination to see whether it provides protection, and if so what this tells us of the biology of HIV transmission.

